# The longitudinal follow-up of a newly proposed OCTA imaging finding (SSPiM) and the importance of it as a new biomarker for treatment response in diabetic macular edema

**DOI:** 10.1007/s00417-024-06457-2

**Published:** 2024-03-26

**Authors:** Gülsüm Genç, Özge Yanık, Sibel Demirel, Figen Batioglu, Emin Özmert

**Affiliations:** Department of Ophthalmology, Ankara University Faculty of Medicine, Ankara, Turkey

**Keywords:** Diabetic macular edema, Optical coherence tomography angiography, OCT biomarker, OCTA biomarker, Suspended scattering particles in motion, Hyperreflective fluid, Treatment resistance

## Abstract

**Purpose:**

This study aimed to evaluate the frequency of SSPiM (suspended scattering particles in motion), systemic risk factors, ocular findings, progression characteristics, and treatment response in diabetic retinopathy (DR) patients.

**Methods:**

In this prospective study, a total of 109 eyes of 109 patients with diabetic macular edema (DME) were included. Demographic characteristics and systemic data of the patients were recorded. In addition to a detailed ophthalmological examination, optical coherence tomography (OCT) and OCT angiography (OCTA) imaging were performed. According to the OCTA images, the patients were divided into two categories: SSPiM detected (SSPiM +) and undetected (SSPiM −). The patients were followed up at 0, 3, and 6 months. Treatment responses at 6 months in treatment-administered patients with and without SSPiM were examined.

**Results:**

The frequency of SSPiM in DME cases was found to be 34.9%. No significant correlation was found between SSPiM and demographic characteristics, systemic, and biochemical parameters (*p* > 0.05). It was observed that SSPIM was most frequently localized in the outer nuclear layer adjacent to the outer plexiform (81.6%). SSPiM appearance disappeared in 7 (19.4%) of 36 patients with SSPiM who had regular follow-up for 6 months. In 4 (11.1%) of these seven patients, hard exudate plaques developed in the areas where SSPiM disappeared. Regarding treatment response at 6 months, the decrease in CMT was statistically significantly lower in the SSPiM group compared to cases without SSPiM.

**Conclusion:**

SSPiM is a finding seen in approximately one-third of DME patients and may adversely affect the response to the treatment.

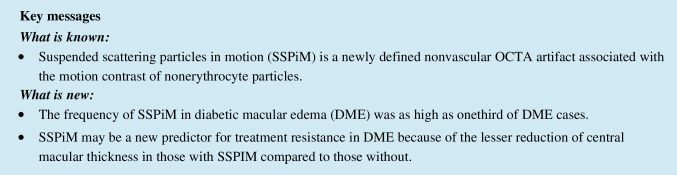

## Introduction

Diabetic macular edema (DME) is among the leading causes of diabetes-related vision loss and can occur in any stage of diabetic retinopathy (DR) [1]. It is seen at rates of 3% in mild nonproliferative DR (NPDR), 38% in severe NPDR, and 71% in proliferative DR (PDR) [2]. The accumulation of edema in the macular region despite the diffuse impairment of the blood-retinal barrier in DR may be attributable to factors such as its high metabolic activity and cell density, loose intercellular junctions in the outer plexiform layer, and low fluid absorption due to the central avascular zone [3].

OCT angiography (OCTA) is a new, noninvasive, high-resolution functional imaging method that detects and processes the motion contrast of intravascular red blood cells to create 3D and cross-sectional images of blood flow in the retinal and choroidal vasculature [4]. Changes in the phase and amplitude of the signals are evaluated mathematically to yield a decorrelation signal [5]. Any change in this tissue movement is attributed to the blood flow within the tissue [6]. OCTA in DR has enabled enlargement and irregularity of the foveal avascular zone, enlargement of the perifoveal intercapillary area, and areas of capillary nonperfusion to be defined and monitored [7–9]. OCTA can also detect early microvascular changes, changes in the superficial/deep retinal vasculature (due to its quantitative measurement feature), and diabetic choroidal pathology in DR patients [10, 11]. Even if OCTA is a promising technique to evaluate non-perfusion areas in vascular diseases, OCTA is susceptible to artifacts. Nonvascular pseudo-flow signals in areas of intraretinal hard exudates and macular drusen have been reported previously [12]. Projection artifact is likely the causative process generating these false positive blood flow signals on these hyperreflective structures [12]. Furthermore, in these reflective regions, scanner light coming to the retina is scattered because of small eye movements and is interpreted by the instrument as a flow signal. Unlike the fluid and red blood cells flowing at a certain rate through the retinal vessels, static accumulations in the intraretinal fluid do not generate a flow signal on OCTA [13]. Recently, there have been reports of nonvascular flow signals on OCTA corresponding to areas of intraretinal fluid on OCT in some cases of macular edema secondary to vascular diseases [14, 15]. SSPiM is a newly defined nonvascular OCTA artifact associated with the motion contrast of non-erythrocyte particulate matter resulting from the leakage of serum and protein from vessels due to blood-retinal barrier breakdown in all exudative maculopathies, especially DR [16]. Instead of hyporeflective cystic spaces on OCT, there are hyperreflective fluid-filled spaces corresponding to SSPiM signals. Scattered lipoprotein and/or protein content floating in these spaces also cause motion contrast [17, 18]. After the introduction of these intraretinal cysts with homogenous hyperreflectivity, which were found to generate flow signals on OCTA due to a phenomenon called SSPiM in diabetes, it was reported as an imaging finding among other vascular diseases [16].

Although the structural OCT features of DME have been extensively studied, these cystic spaces that have internal reflectivity and produce flow signals on OCTA as false flow signals are still not fully understood, and there is no consensus on their interpretation [19, 20]. SSPiM, detected noninvasively by OCTA examination, is a newly defined biomarker that can be incorporated into DR follow-up and is expected to increase in importance in the future. However, there has been inadequate research into the prevalence, etiology, and clinical significance of this nonvascular pseudo-flow signal on OCTA. The aim of this study was to evaluate the frequency of SSPiM in DME, the fate of SSPiM during follow-up, the associated risk factors of SSPiM in patients with DR, and whether it can be a biomarker for the macular perfusion or for the treatment response of anti-VEGF treatment.

## Materials and methods

### Patient selection

This prospective controlled study included 109 consecutive patients with DME who were followed up for DR in the Retina-Vitreous Unit at the Department of Ophthalmology of Ankara University Faculty of Medicine between September 2020 and April 2021. Among these 109 patients, 45 had bilateral DME. In cases of bilateral DME, one eye was randomly selected, and 109 eyes of these 109 patients were included in the study.

Inclusion criteria were age over 18 years, diagnosis of DME secondary to diabetes mellitus, regular follow-up in the endocrine outpatient clinic, and an OCTA image quality of 7/10 or higher. The eyes were categorized into groups based on the detection of SSPiM (SSPiM + group) or its absence (SSPiM − group) using OCTA. SSPiM was defined as extra-vascular motion signals on both cross-sectional and en-face OCTA images [16]. This signal was distinguishable from other types of projection artifacts by features such as corresponding (on B-scan) regular and oval-shaped intraretinal cystic lesions containing hyperreflective material inside them, which is markedly different from irregularly shaped hard exudates or other intraretinal collections with back shadowing.

Exclusion criteria were the presence of any additional eye disease such as uveitis, glaucoma, retinal vein occlusion, age-related macular degeneration, or vitreoretinal interface pathologies (e.g., vitreomacular adhesion, vitreomacular traction, epiretinal membrane, macular hole), media opacity that would prevent fundus imaging, history of any intraocular surgery in the last 6 months, and history of focal/grid laser treatment in the last 3 months.

Detailed ophthalmological (cataract surgery, total number and type of intravitreal injections, total number of laser treatments) and systemic (type and duration of diabetes mellitus (DM), type and duration of DR, antidiabetic drugs used, duration of insulin use, presence and duration of hypertension, hyperlipidemia, chronic kidney disease, coronary artery disease, cerebrovascular disease, obstructive sleep apnea syndrome, asthma/chronic obstructive pulmonary disease, and smoking) histories were obtained from all participants.

Patients in the study attended regular follow-up in the endocrine clinic, and their biochemical parameters (HbA1c, fasting blood glucose, hemoglobin, blood urea nitrogen, and creatine) were evaluated at 0, 3, and 6 months. However, 15 patients discontinued follow-up for various reasons during the study period and did not have data from months 3 and 6. These patients’ baseline data were evaluated, but they were excluded from analyses of follow-up data.

Best corrected visual acuity (BCVA) was measured using the Early Treatment of Diabetic Retinopathy Study (ETDRS) chart. All patients underwent slit-lamp anterior segment examination, ocular blood pressure measurement, and dilated fundus examination with non-contact lenses. DR was classified as NPDR (mild, moderate, or severe) and PDR according to the ETDRS grading system. Patients with very severe NPDR were included in the severe NPDR group.

The presence of DME and CMT were evaluated with spectral domain OCT (SD-OCT). SD-OCT scans were obtained using Spectralis HRA + OCT device (Heidelberg Engineering, Heidelberg, Germany). This device can acquire 85,000 A-scans per second with an axial resolution of 7 μm, a lateral resolution of 14 μm, and a central wavelength of 870 nm. The scan protocol consisted of a 30° × 20° OCT volume scan in HS mode and 512 A-scans per B-scan using automatic real-time tracking (ART) mode averaging 25 B-scans per final B-scan. DME type was determined according to the most recent International Council of Ophthalmology classification as non-center-involving DME (retinal thickening in the macula that does not involve the 1-mm central subfield zone) or center-involving DME (retinal thickening in the macula that involves the 1-mm central subfield zone) [21]. Although there are differing opinions in the literature regarding the central foveal thickness value used to define central-involving edema, it has been argued that a value of at least 225–270 μm should be used [14, 15]. In our study, we defined this value as 250 μm.

OCTA measurements were obtained using the AngioVue OCTA software of RTVue XR Avanti (Optovue, Inc., Fremont, CA). This 70,000 Hz (840-nm wavelength) system is an SD-OCT based system and uses the split spectrum amplitude decorrelation angiography algorithm. It provides 5 μm axial resolution and 15 μm lateral resolution. We obtained 6 mm × 6 mm macular OCTA scans with a high scanning density (400 × 400). The following evaluations were made on 6 × 6-mm images: the presence of SSPiM, the presence of hyperreflective fluid and material associated with SSPiM, and the location of SSPiM-associated hyperreflective fluid (outer nuclear layer and inner nuclear layer). Two senior retina specialists (E.Ö., F.B.) who had more than 30 years of working experience in diagnosing and treating retinal disorders blindly evaluated the OCTA images regarding SSPiM presence. Disagreements between them were openly adjudicated by another experienced retina specialist (SD). Color fundus photography (Clarus; Carl Zeiss, Dublin, USA) and/or multicolor imaging by confocal scanning laser ophthalmoscopy (Spectralis HRA + OCT) were examined for the development of hard exudates during the follow-up period.

The built-in software “AngioAnalytics” of the Optovue Avanti RTVue XR device was used to measure quantitative parameters automatically including foveal avascular zone (FAZ) area (mm2) and vessel density percentages (VD%) in the superficial and deep capillary plexuses during the follow-up period. Superficial and deep vessel densities were calculated separately in the foveal (central 1-mm diameter), parafoveal (1- to 3-mm diameter ring), and perifoveal (3- to 6-mm diameter ring) regions. Nonperfused areas of the superficial capillary plexus (mm2) were manually marked using the device’s “AngioAnalytics” feature and calculated automatically by the software. The repeatability and reproducibility of quantitative measurements using the Avanti systems’ in-built analytics were reported to be relatively high by previous publications [22, 23].

Patients who required treatment during follow-up were treated, and their CMT values at baseline and 6 months were compared to evaluate treatment response. A decrease of 10% or more from baseline CMT was considered a response to treatment. Our treatment protocol for patients with DME involved administering the first three loading doses of anti-VEGF, followed by a pro-re-nata regimen. In cases where patients showed an inadequate response to anti-VEGF treatment, we switched to an intravitreal 0.7 mg dexamethasone implant (Ozurdex™, Allergan®), if they were pseudophakic and had normal intraocular pressure. Additionally, for patients exhibiting extrafoveal edematous areas after anti-VEGF treatment, a subthreshold 577 nm yellow wavelength micropulse laser (Supra Scan, Quantel Medical, Cedex, France) was applied as an adjunctive therapy.

### Statistical analysis

Statistical analyses of the data were performed using the IBM SPSS version 20.0 software package (IBM Corp., Armonk, NY, USA). In patients with bilateral edema, one eye was randomly selected using a computer-generated random sequence to ensure the accuracy of the statistical analysis and prevent selection bias. The normality of data distributions was assessed using the Kolmogorov–Smirnov test. Normally distributed numerical variables were expressed as mean ± standard deviation, nonnormally distributed numerical variables as median (minimum–maximum), and categorical variables as frequency (percentage). For comparisons between two independent groups, numerical variables were compared using independent-samples *t*-test if normally distributed and Mann–Whitney *U* test if nonnormally distributed. For comparisons of more than two independent groups, numerical variables were compared using one-way analysis of variance (ANOVA) if normally distributed and Kruskal–Wallis test if nonnormally distributed. Comparisons of numerical values between two dependent groups were done using *t*-test if normally distributed and Wilcoxon test if nonnormally distributed. Comparisons of categorical variables were evaluated with chi-square analysis. Cohen’s kappa coefficient was calculated to quantify the intergrader agreement for SSPiM detection. A two-tailed *p* < 0.05 was considered statistically significant.

## Results

### Demographic and clinical examination data

The demographic, systemic, and ophthalmological data of the patients are shown in Table 1. In the 109 eyes of 109 patients included in the study, SSPiM was detected in 38 (34.9%) on baseline OCTA images. For the detection rate of SSPiM, Cohen’s kappa coefficient between two graders was almost perfect (0.939). The SSPiM + group included 13 women (34.2%) and 25 men (65.8%). The SSPiM − group included 31 women (43.7%) and 40 men (56.3%). The mean age of the patients with SSPiM was 62.39 ± 8.6 years. Age and sex distribution were similar in the SSPiM + and SSPiM − groups (*p* = 0.338 and *p* = 0.843, respectively).

**Table 1:** The patients’ demographic, systemic, and ophthalmologic data (*n* = 109)

Variable	SSPiM − group (*n* = 71)	SSPiM + group (*n* = 38)	*p*
Age	62.39 ± 8.63	62.05 ± 8.39	0.843*
Sex			
Female	31 (43.7)	13 (34.2)	0.338 ‡
Male	40 (56.3)	25 (65.8)	
Side			
Right	37 (52.1)	19 (50.0)	0.833 ‡
Left	34 (47.9)	19 (50.0)	
BMI (kg/m^2^)	27 (25.4–29)	28 (26.22–29)	0.430†
DM type			
1	2 (2.8)	2 (5.3)	0.610 ‡
2	69 (97.2)	36 (94.7)	
DM duration (years)	20 (12–24)	18 (10–22)	0.475†
DR duration (years)	3 (2–6)	2.5 (1–6)	0.307†
DR type			
Mild NPDR	6 (8.4)	0 (0.0)	0.062 ‡
Moderate NPDR	19 (26.8)	18 (47.4)	
Severe NPDR	19 (26.8)	10 (26.3)	
PDR	27 (38)	10 (26.3)	
Diabetic neuropathy	33 (46.5)	16 (42.1)	0.662 ‡
Diabetic nephropathy	22 (31.0)	10 (26.3)	0.610 ‡
Comorbidities			
CAD	25 (35.2)	16 (42.1)	0.479 ‡
CVA	2 (2.8)	2 (5.3)	0.610 ‡
HT	53 (74.6)	31 (81.6)	0.412 ‡
HT duration (years)	12 (9–20)	11 (6–19)	0.576†
HPL	28 (39.4)	18 (47.4)	0.424 ‡
CKD	14 (19.7)	6 (15.8)	0.614 ‡
OSAS	2 (2.8)	1 (2.6)	1.000 ‡
Asthma/COPD	8 (11.3)	3 (7.9)	0.744 ‡
Smoking	18 (25.4)	11 (28.9)	0.686 ‡
Antidiabetic drug			
Oral antidiabetic	15 (21.1)	13 (34.2)	0.193 ‡
Insulin	22 (31.0)	13 (34.2)	
Oral antidiabetic + insulin	34 (47.9)	12 (31.6)	
Insulin duration (years)	10 (6–15)	7 (6–11)	0.406†
History of cataract surgery			
Phakic	32 (45.1)	25 (65.8)	**0.007‡**
Pseudophakic	39 (54.9)	13 (34.2)	
Total number of anti-VEGF injections	8 (3–13)	6 (4–10)	0.228†
BCVA (LogMAR)	0.3 (0.1–0.4)	0.22 (0.1–0.4)	0.381†
DME type			
Non-center-involving	17 (23.9)	16 (42.1)	**0.049 ‡**
Center-involving	54 (76.1)	22 (57.9)	
IOP (mmHg)	16 (13–19)	15 (13–18)	0.265†
Biochemical Parameters			
HbA_1c_	8.66 ± 1.86	8.80 ± 1.60	0.701*
Fasting blood glucose	166 (140–202)	158.5 (133–201.25)	0.672†
Hemoglobin	13 (12–13.9)	13.2 (11.95–14.55)	0.335†
Blood urea nitrogen	23 (18–35)	20 (15.15–25.25)	**0.041†**
Creatinine	0.84 (0.7–1.13)	0.88 (0.69–1.28)	0.731†

Baseline data are given for BCVA, DME type, IOP, and biochemical parameters

*SSPiM* suspended scattering particles in motion, *n* number of patients, *BMI* body mass index, *DM* diabetes mellitus, *DR* diabetic retinopathy, *NPDR* nonproliferative DR, *PDR* proliferative DR, *CAD* coronary artery disease, *CVA* cardiovascular accident, *HT* hypertension, *HPL* hyperlipidemia, *CKD* chronic kidney disease, *OSAS* obstructive sleep apnea syndrome, *COPD* chronic obstructive pulmonary disease, *BCVA* best corrected visual acuity, *DME* diabetic macular edema, *IOP* intraocular pressure

^*^Independent *t*-test (mean ± SD); †Mann–Whitney *U* test (median [Q1–Q3]); ‡Chi-square test (*n* [%])

Four (3.7%) of the patients had type 1 DM, and 105 (94.7%) had type 2 DM. There was no significant difference in the distribution of DM type between the two groups (*p* = 0.610). The median duration of DM was 18 years (range, 10–22) in the SSPiM + group and 20 years (range, 12–24) in the SSPiM − group. There was no statistically significant difference between the groups in terms of the duration of diabetes (*p* = 0.475).

The mean HbA1c level was 8.80% ± 1.60% in the SSPiM + group and 8.66% ± 1.86% in the SSPiM − group (*p* = 0.701). The two groups showed no statistically significant differences in terms of the prevalence of systemic diseases. Hypertension was the most common comorbidity in both groups and was present in 31 (81.6%) of the patients in the SSPiM + group.

In the SSPiM + group, ten patients (26.3%) had mild NPDR, 18 (47.4%) had moderate NPDR, and ten (26.3%) had severe NPDR. In the SSPiM − group, six patients (8.5%) had mild NPDR, 19 (26.8%) had moderate NPDR, 19 (26.8%) had severe NPDR, and 27 (38%) had PDR. However, the difference in the distribution of DR types between the two groups did not reach statistical significance (*p* = 0.062).

### Anatomical characteristics of SSPiM

**Fig. 1:**
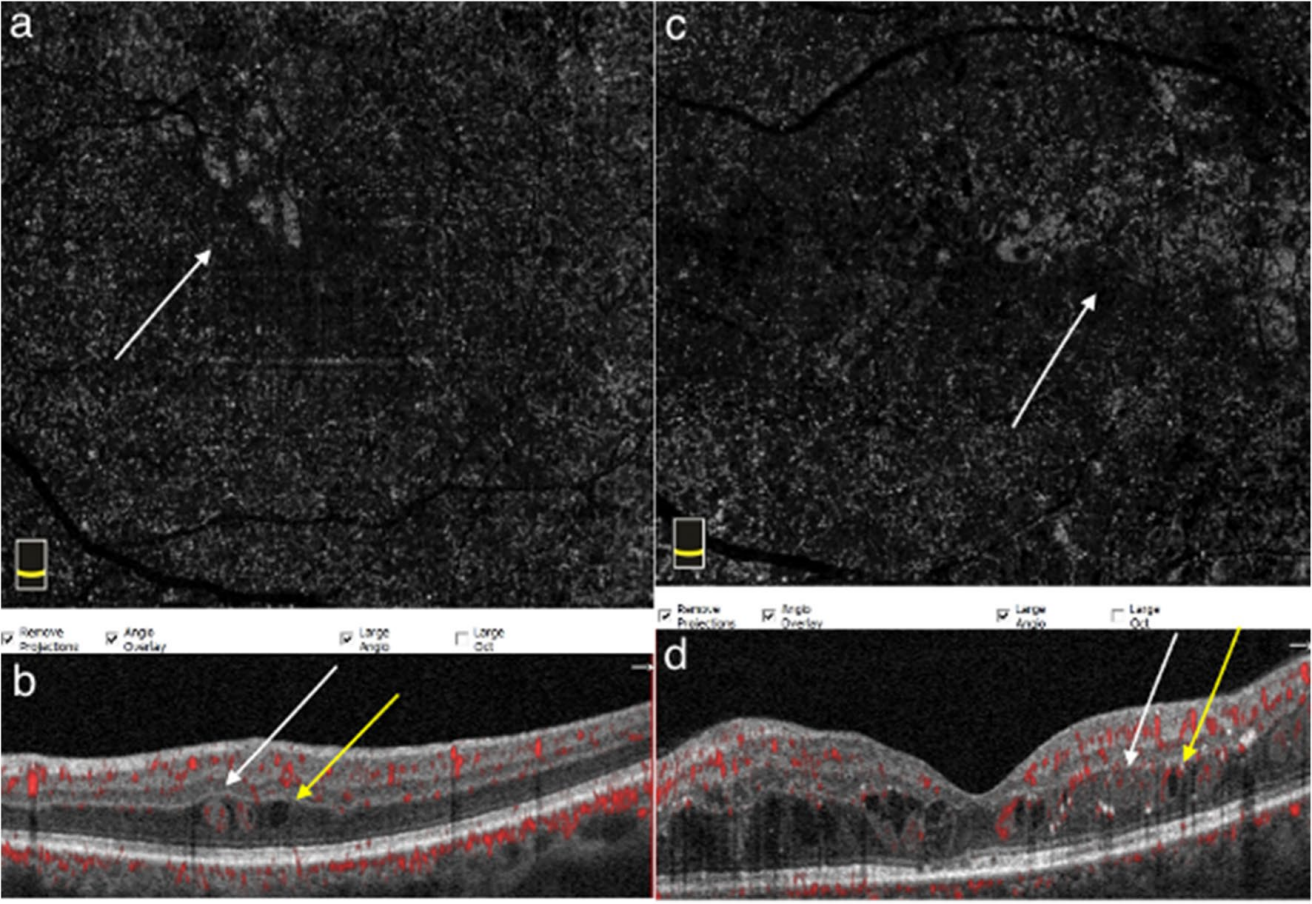
SSPiM appearance in two different cases. Case 1: A 58-year-old man with type 2 DM for 20 years. **a** On the outer retinal slab of en-face OCTA (upper left), the pseudo-flow signal extending from the center of the FAZ to the periphery is indicated with a white arrow. **b** Cross-sectional B-scan OCT image with superimposed OCTA flow signal (lower left) shows hyperreflective fluid with no hyperreflective material at the margin and a nonvascular pseudo-flow signal shown in red (white arrow), as well as hyporeflective fluid with no flow signal (yellow arrow). Case 2: A 53-year-old man with type 2 DM for 15 years. **c** On the outer retinal slab of en-face OCTA (upper right), the pseudo-flow signal extending from the center of the FAZ to the periphery is indicated with a white arrow. **d** Cross-sectional B-scan OCT image with superimposed OCTA flow signal (lower right) shows hyperreflective fluid accompanied by hyperreflective material and a nonvascular pseudo-flow signal shown in red (white arrow), as well as hyporeflective fluid with no flow signal (yellow arrow)

The anatomical features of SSPiM on OCTA were noted. SSPiM signals were localized to the FAZ margin (vascular/avascular junction) or center. SSPiM was associated with corresponding hyperreflective fluid in all cases. SSPiM was also associated with hyperreflective material in 34 eyes (89.5%). The SSPiM-related hyperreflective fluid was located in the outer nuclear layer adjacent to the outer plexiform layer in 31 eyes (81.6%) and in the inner nuclear layer in seven eyes (18.4%) (Fig. 1).

**Fig. 2:**
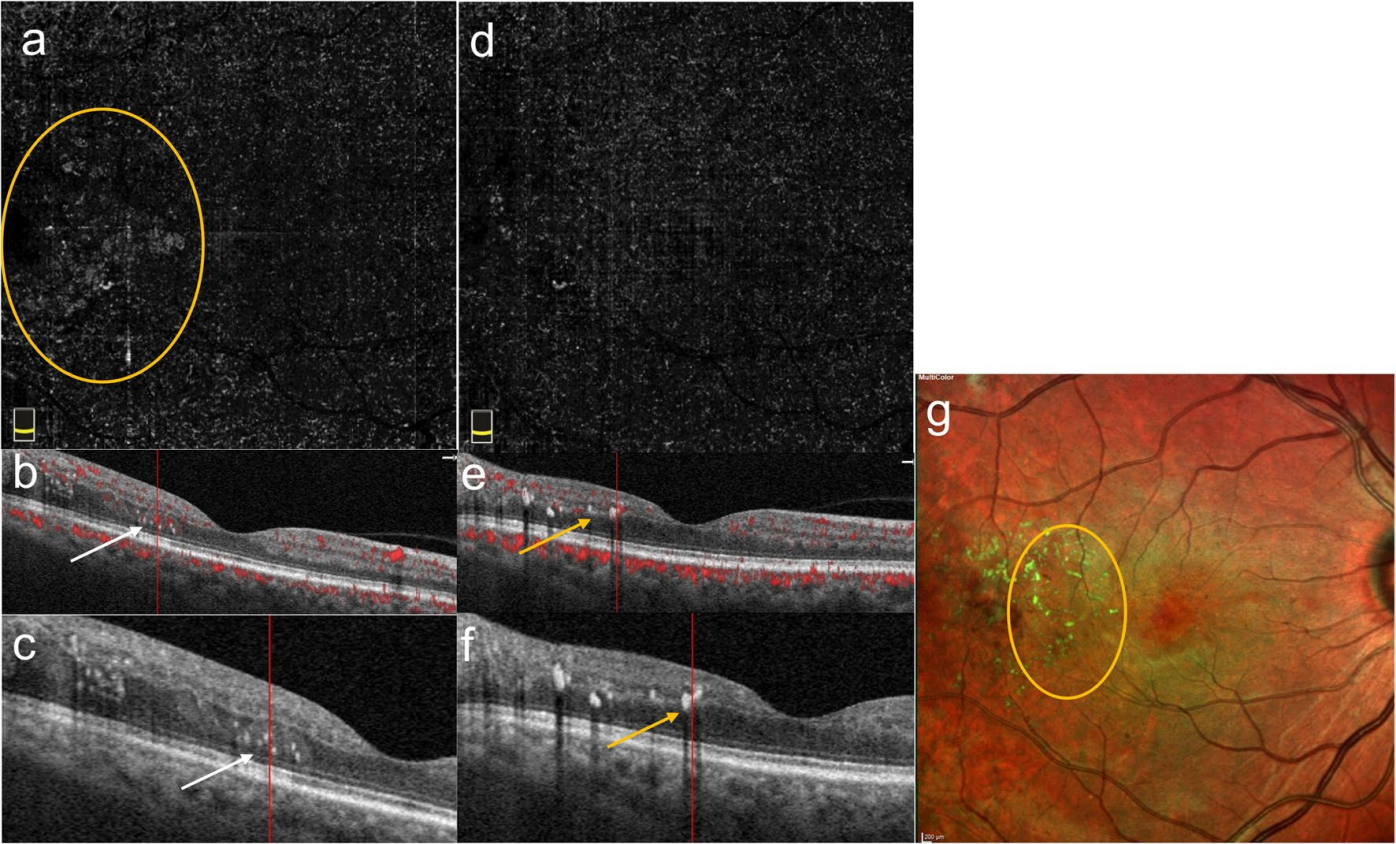
Conversion of SSPiM to hard exudate over time. Case 3: A 45-year-old man with type 2 DM for 7 years. **a** En-face OCTA (upper left) shows hyperreflectivity corresponding to the pseudo-flow signal around the FAZ (yellow circle). **b** B-scan image with superimposed OCTA flow signal (middle left) shows a nonvascular pseudo-flow signal shown in red. **c** Baseline structural B-scan OCT image (lower left) shows hyperreflective fluid accompanied by hyperreflective material (white arrow). At 6-month follow-up, **d** the pseudo-flow signal is no longer detected on en-face OCTA. However, the appearance of hard exudates was observed at the location of the SSPiM. **e** OCTA-superimposed B-scan sections (yellow arrows) **f** Structural B-scan sections (yellow arrows). **g** Multicolor imaging (yellow circle)

**Fig. 3:**
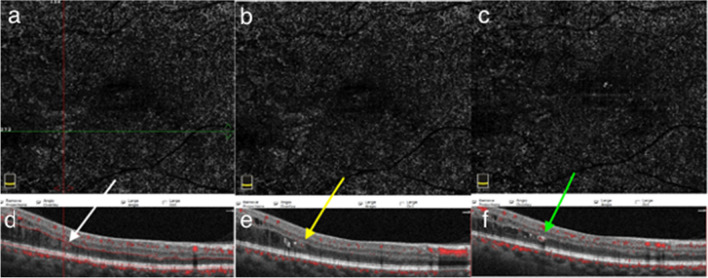
Conversion of SSPiM to hard exudate over time. Case 4: A 65-year-old man with type 2 DM for 21 years and PDR for 3 years. The patient had a history of panretinal laser photocoagulation, micropulse laser, and multiple intravitreal anti-VEGF injections. At 5-month follow-up, the en-face OCTA images show a decrease and disappearance of the pseudo-flow signal. **a** Initial en-face OCTA images. **b** Three-month en-face OCTA images. **c** Five-month en-face OCTA images. **d** B-scan OCT images superimposed with OCTA flow signals show hyperreflective fluid (white arrow). **e** In the outer nuclear layer adjacent to the outer plexiform layer at baseline, accumulation of hyperreflective material (yellow arrow) at the edge of the hyperreflective fluid at 3 months. **f** The appearance of a hyperreflective retinal spot larger than 30 µm with back shadowing (green arrow) due to the coalescenc of hyperreflective material at 5 months

In seven patients (19.4%), SSPiM findings disappeared at approximately 5 months (range, 4–6 months). In four (11.1%) of these patients, the hyperreflective materials increased in quantity and coalesced to form hard exudates (Figs. 2 and 3). In the other three patients (8.3%), SSPiM resolved without the formation of any hyperreflective material or hard exudate at the edge of the hyperreflective fluid. In some eyes, SSPiM disappeared from one site but was later detected in another area during follow-up. The clinical and anatomical characteristics of patients with SSPiM are examined according to DR type in Table 2.

**Table 2:** Anatomical characteristics of SSPiM according to DR type in the SSPiM + group

Variable SSPiM + group	Moderate NPDR (*n* = 18)	Severe NPDR (*n* = 10)	PDR (*n* = 10)	
SSPiM-associated HRF	18 (100)	10 (100)	10 (100)	
SSPiM-associated HRM				
No	2 (11.1)	1 (10.0)	1 (10.0)	1.000‡
Yes	16 (88.9)	9 (90.0)	9 (90.0)	
HRF location				
Outer layer	14 (77.8))	9 (90.0)	8 (80.0)	0.718‡
Inner nuclear layer	4 (22.2)	1 (10.0)	2 (20.0)	

*SSPiM* suspended scattering particles in motion, *n* number of patients, *HRF* hyperreflective fluid, *HRM* hyperreflective material, *Outer layer* the outer nuclear layer adjacent to the outer plexiform layer

^‡^Chi-square test (*n* [%])

## Changes during follow-up

### Comparison of initial data in the SSPiM + and SSPiM − groups

Anatomically, there was no significant difference between the groups in terms of baseline CMT (*p* = 0.962) (Table 3).

**Table 3:** Initial quantitative data from the SSPiM + and SSPiM − groups

Variable	SSPiM − group (*n* = 71)	SSPiM + group (*n* = 38)	*p*
BCVA (LogMAR)	0.3 (0.1–0.4)	0.22 (0.1–0.4)	0.381†
CMT (μm)	310 (294–357)	318.5 (285–358.75)	0.962†

*SSPiM* suspended scattering particles in motion, *n* number of patients, *BCVA* best corrected visual acuity, *CMT* central macular thickness

^†^Mann–Whitney *U* test (median [Q1–Q3])

### Comparison of final data in the SSPiM + and SSPiM − groups

Although the study plan involved 0-, 3-, and 6-month assessments, 15 of the 109 DME patients initially included did not continue follow-up. Therefore, the follow-up analysis includes only the 94 DME patients with data from 0, 3, and 6 months. The final BCVA was similar in the two groups (*p* = 0.548).

At 6 months, CMT was higher in the SSPiM + group, which was statistically significant (*p* = 0.027). Final (6-month) quantitative data from the SSPiM + and SSPiM − groups are shown in Table 4.

**Table 4:** Final (6-month) quantitative data from the SSPiM + and SSPiM − groups

Variable	SSPiM − group (*n* = 58)	SSPiM + group (*n* = 36)	*p*
BCVA (LogMAR)	0.21 (0.1–0.2)	0.25 (0.1–0.4)	0.548†
CMT (μm)	276 (259.75–322)	320 (267–371.75)	0.027†

*SSPiM* suspended scattering particles in motion, *n* number of patients, *BCVA* best corrected visual acuity, *CMT* central macular thickness

^†^Mann–Whitney *U* test (median [Q1–Q3])

### Comparison of treatment response according to the presence of SSPiM

During the 6-month follow-up period, 18 (50%) of the 36 patients in the SSPiM + group received only intravitreal anti-VEGF therapy, seven (19.5%) received intravitreal anti-VEGF and subthreshold 577 nm yellow wavelength micropulse laser therapy, and two (5.5%) received intravitreal 0.7 mg dexamethasone implants and subthreshold 577 nm yellow wavelength micropulse laser. In the SSPiM − group, 40 (68.9%) of the 58 patients received only intravitreal anti-VEGF therapy, three (5.2%) received intravitreal 0.7 mg dexamethasone implants, four (6.9%) received intravitreal anti-VEGF and intravitreal 0.7 mg dexamethasone implants, and three (5.2%) received intravitreal anti-VEGF and subthreshold 577 nm yellow wavelength micropulse laser therapy. None of the patients in this group were treated with a combination of intravitreal 0.7 mg dexamethasone implant and subthreshold 577 nm yellow wavelength micropulse laser therapy. No patients in either group were treated with subthreshold 577 nm yellow wavelength micropulse laser therapy alone. Nine patients (25%) in the SSPiM + group and 8 (13.8%) in the SSPiM − group were followed up for 6 months without treatment (Table 5).

**Table 5:** Treatments received by patients in the SSPiM + and SSPiM − groups during 6-month follow-up

Variable	SSPIM − group (*n* = 58)	SSPIM + group (*n* = 36)
Intravitreal anti-VEGF monotherapy	40 (68.9)	18 (50)
Intravitreal steroid monotherapy	3 (5.2)	0
Micropulse laser (MPL) monotherapy	0	0
Intravitreal anti-VEGF + intravitreal steroid therapy	4 (6.9)	0
Intravitreal anti-VEGF + MPL therapy	3 (5.2)	7 (19.5)
Intravitreal steroid + MPL therapy	0	2 (5.5)
Followed without treatment	8 (13.8)	9 (25)

*SSPiM* suspended scattering particles in motion, *n* (%)

Treatment response to intravitreal anti-VEGF monotherapy was evaluated in the SSPiM + and SSPiM − groups. Response was defined as a decrease in CMT of 10% or more from baseline. The treatment response rate was lower in the SSPiM + group compared to the SSPiM − group (33.3% vs. 62.5%, *p* = 0.039). Patients in the SSPiM + and SSPiM − groups who received intravitreal anti-VEGF monotherapy did not differ statistically in terms of DME type, baseline CMT, anti-VEGF agent received, or number of anti-VEGF injections (*p* > 0.05 for all). However, final CMT was significantly higher in the SSPiM + group compared to the SSPiM − group (*p* = 0 0.019) (Table 6).

**Table 6:** Comparison of treatment response to intravitreal anti-VEGF monotherapy at 6 months in the SSPiM + and SSPiM − groups

Variable	SSPIM − group (*n* = 40)	SSPIM + group (*n* = 18)	*p*
DME type			0.261‡
Center-involving	38 (80.9)	18 (69.2)	
Non-center-involving	9 (19.1)	8 (30.8)	
CMT initial (μm)	316.5 (300.25–369.75)	330.5 (300.75–387.75)	0.501†
CMT final (μm)	280 (262.75–336.25)	326 (308.75–375.75)	**0.019†**
Number of anti-VEGF injections	3 (2–3)	3 (2–4.25)	0.317†
Anti-VEGF type			0.521‡
Aflibercept	16 (40)	7 (38.9)	
Ranibizumab	13 (32.5)	3 (16.7)	
Bevacizumab	9 (22.5)	6 (33.3)	
Bevacizumab + aflibercept	2 (5)	2 (11.1)	
Treatment response*	25 (62.5)	6 (33.3)	**0.039‡**

*SSPiM* suspended scattering particles in motion, *n* number of patients, *DME* diabetic macular edema, *CMT* central macular thickness

^†^Mann–Whitney *U* test (median [Q1–Q3]); ‡chi-square test (*n* [%]); *defined as a decrease of 10% or more in CMT from baseline to 6 months

## Discussion

In this study, the overall prevalence of SSPiM in patients with various stages of DME was 34.9%. Analyses to identify potential risk factors revealed no significant relationships between the presence of SSPiM and age, sex, systemic comorbidities, diabetes duration, or other biochemical serum parameters. Among the patients with SSPiM, hyperreflective material was observed at the edge of the hyperreflective fluid corresponding to SSPiM in 89.5%. SSPiM was most commonly located in the outer nuclear layer adjacent to the outer plexiform layer (81.6%) and was either associated with the development of hard exudate during follow-up or resolved without the formation of hard exudate. Of the patients who received anti-VEGF monotherapy during follow-up, we noted a lower treatment response rate at 6 months in those with SSPiM. This suggests that patients may be more resistant to treatment in the presence of SSPiM.

The presence of SSPiM has also been demonstrated in pathologies such as DR [16, 24, 25], radiation retinopathy, neovascular age-related macular degeneration, retinal arterial microaneurysm [16], retinal vein occlusion [16, 26], Coats disease [27], and perifoveal abnormal vascular complex [28]. In a study based on single-center data, the prevalence of SSPiM in DME patients was reported to be 27.8%. While SSPiM was not detected in patients with mild DR, its prevalence was 66.7% in those with moderate NPDR, 26.3% in those with severe NPDR, and 24.1% in those with PDR [16]. Consistent with the literature, SSPiM was detected in over one-third of DME patients in our study (34.9%). Similarly, SSPiM was not detected in any of the patients with mild NPDR, while the rate was 48.6% in those with moderate NPDR, 34.5% in those with severe NPDR, and 27% in those with PDR. Although a combination of lipid and protein-containing macromolecules is believed to cause SSPiM signals, it is not clear exactly what types of particles in the intraretinal fluid are detected by OCTA in vivo. It has been reported in the literature that high lipid levels in the blood are associated with increased hard exudates in DR [29]. Another study showed that high low-density lipoprotein values were associated with hyperreflective material on OCT [30]. This information suggests that extravascular lipids may contribute to SSPiM and subsequently to the formation of the hyperreflective material and hard exudate associated with SSPiM [16]. Although lipid levels in the edema may be a factor in its appearance, SSPiM was not found to be associated with systemic hyperlipidemia. In a study by Ahn et al. [31] examining this relationship, we observed a similar rate of hyperlipidemia in patients with and without SSPiM. In addition, SSPiM was not associated with factors such as comorbidities, HbA1c level, or diabetes duration in our study. This may be because the low incidence of this imaging feature requires a large sample size to detect statistical significance. Even if we could not find any systemic relationship, the occurrence of SSPiM in the later stage of DR may be an indirect indicator of blood-retinal barrier breakdown and the degree or severity of vascular involvement.

When the location of SSPiM was evaluated, we noted that SSPiM foci were found in the FAZ center or margin, which is the vascular/avascular junction. Hyperreflective fluid was observed in the areas corresponding to SSPiM. Consistent with the literature, these hyperreflective fluid regions were mostly located in the outer nuclear layer adjacent to the outer plexiform layer [16, 25, 31]. In our patients with SSPiM, the hyperreflective fluid was in the outer nuclear layer adjacent to the outer plexiform layer in 81.6% of cases and in the inner nuclear layer in only 18.4% of cases. The outer part of the retina is known to be most commonly and severely affected by edema due to its anatomical characteristics [32, 33]. These results are consistent with our findings that SSPiM-associated hyperreflective fluid preferentially localizes to the outer nuclear layer adjacent to the outer plexiform layer. In the study by Kashani et al., 88.2% of the hyperreflective fluid associated with SSPiM was in Henle’s layer, and 19.7% was in the inner nuclear layer [16]. Murakami et al. observed in their study of diabetic eyes that decorrelation signals were not accompanied by hyperreflective fluid in the inner nuclear layer [34]. In the literature, similar to our study, SSPiM has not been reported in any location corresponding to subretinal fluid [16]. The absence of SSPiM in the subretinal fluid may be explained by findings from a study of exudative retinopathies indicating that particulate matter can be cleared from the subretinal space more effectively than from the intraretinal space [35].

Another finding in our study was the anatomical relationship between hyperreflective material and hyperreflective fluid. Hyperreflective material was observed at the margins of areas of hyperreflective fluid associated with SSPiM in 89.5% of cases. There is still no consensus on the origin and importance of these hyperreflective materials that line the cystoid cavities seen in various exudative maculopathies. It also remains unclear whether this hyperreflective material consists of intracellular or extracellular residue, or both. Gelman et al. in chronic exudative maculopathies and Ajay et al. in DME patients reported that these hyperreflective materials were arranged along the inner wall of the cystoid spaces in the outer plexiform layer. They called the finding in this OCT the pearl necklace sign. The authors speculate that the hyperreflective material is composed of lipoproteins or lipid-laden macrophages. The pearl necklace sign is thought by the authors to be a precursor to hard exudates [36, 37].

OCTA generally distinguishes between nonperfused areas and vascular areas, but nonvascular decorrelation signals or artifacts may hinder this distinction [38]. In the baseline comparison of patients with and without SSPiM, those with SSPiM had significantly larger nonperfused areas on OCTA. In the literature, it has been reported that measurements of nonperfused areas on OCTA are larger in the presence of cysts and edema [8, 39]. As SSPiM is associated with the breakdown of the blood-retinal barrier, it cannot be distinguished whether an increase in measured nonperfused area is an artifact related to cysts or to ischemic areas. Furthermore, since DME can often cause artifact and segmentation errors in OCTA, there is no consensus regarding the interpretation of any correlation between SSPiM and any OCTA findings.

In seven (19.4%) of the 36 patients with SSPiM who continued regular follow-up for 6 months, the SSPiM signal was found to disappear at an average of 5 months. These patients with DME were on anti-VEGF therapy. Four (11.1%) of these seven patients developed hard exudate plaques corresponding to the hyperreflective material in the areas where the SSPiM had been. In the other three patients (8.3%), SSPiM disappeared without the formation of any hyperreflective material or hard exudate at the edge of the hyperreflective fluid. Hard exudate plaques have been observed to develop over time in DME patients with hyperreflective material after anti-VEGF treatment [40]. Kashani et al. also reported that hard exudate plaques developed in areas that previously created SSPiM signals [16]. Investigation into the features and clinical relevance of SSPiM led to the question of whether it is related to treatment response. Choi et al. reported a poor response to intravitreal bevacizumab therapy in the presence of SSPiM in eyes with branch retinal vein occlusion and cystoid macular edema. In the same study, the number of microaneurysms in the deep capillary plexus was found to be higher in eyes with SSPiM compared to those without SSPiM [26]. In the study by Ahn et al., SSPiM was associated with resistance to treatment in eyes with DME, while SSPiM-associated cysts in the inner nuclear layer showed a better treatment response [31]. In our study, we compared patients with and without SSPiM who received only intravitreal anti-VEGF therapy during follow-up and found that those with SSPiM had a lower treatment response rate (33.3%) than those without SSPiM (62.5%) despite comparable baseline characteristics. Considering that hyperreflective fluid is observed with advanced breakdown of the blood-retinal barrier in DME and other exudative maculopathies, it is plausible that patients with SSPiM may be more resistant to treatment. In our longitudinal study, we have observed that SSPiM areas may turn into hard exudate throughout follow-ups; therefore presence of SSPiM on OCTA, as a precursor to hard exudate, may be an early predictor of treatment resistance. Hard exudate was known to be a cause of treatment resistance resulting in poor visual prognosis [29, 41]. However, these studies were conducted before the era of anti-VEGF therapies and their results cannot be projected to our study due to the difference in the current standard treatment approach in DME. Additionally, in these studies, treatment response was assessed based on visual acuity in contrast to our study in which differences were observed only in CMT between patients with and without SSPiM, with no variation in BCVA.

There are several limitations to our study. The main limitation is the small sample size. Although DR and DME are common in routine ophthalmology practice, SSPiM is not as common, which limited the number of patients in the SSPiM group. Furthermore, as the SSPiM artifact is newly described, there are few studies on SSPiM for comparison in the literature. Studies with longer follow-up will allow an assessment of the utility of SSPiM as a prognostic biomarker in the DME, which is a chronic inflammatory process.

## Declarations

All procedures performed in our study involving human participants were in accordance with the ethical standards of the institutional ethics committee and with the 1964 Helsinki Declaration and its later amendments or comparable ethical standards. Ethical approval for the study was obtained from the Ankara University Faculty of Medicine Ethics Committee (decision number: İ8-488–20, date: 10/09/2020).
